# Prevalence of non-alcoholic fatty liver disease among inflammatory bowel disease patients: a systematic review and meta-analysis

**DOI:** 10.3389/fmed.2025.1517462

**Published:** 2025-07-16

**Authors:** Yuyang Zhao, Jiangbin Wang, He Sun

**Affiliations:** ^1^Department of Gastroenterology, China-Japan Union Hospital of Jilin University, Changchun, Jilin, China; ^2^School of Management Science and Information Engineering, Jilin University of Finance and Economics, Changchun, Jilin, China

**Keywords:** inflammatory bowel disease (IBD), non-alcoholic fatty liver disease (NAFLD), prevalence, risk factors, incidence

## Abstract

**Background:**

Recent studies suggest that individuals with inflammatory bowel disease (IBD) are at a significantly higher risk of developing non-alcoholic fatty liver disease (NAFLD) compared to the general populace. Furthermore, the coexistence of NAFLD is likely to intensify the overall health impact experienced by individuals with IBD.

**Methods:**

This review aims to assess the prevalence of NAFLD and its associated risk factors in IBD patients through systematic analysis. We searched for relevant literature in the PubMed and Medline databases from January 2014 to April 2024 and conducted a meta-analysis to quantitatively synthesize eligible studies, the search criteria were designed to encompass a broad spectrum of research investigating the link between IBD and NAFLD. After completing the literature search, a meticulous screening process was undertaken to filter out studies that did not meet the predefined eligibility criteria.

**Results:**

The analysis encompassed 26 studies, representing a cohort of over 429,550 IBD patients. Our study indicated that the aggregate incidence of NAFLD within this IBD population was 34%, with a 95% confidence interval ranging from 27% to 41%. Additionally, the research highlighted that the likelihood of NAFLD onset is increased in IBD patients with a prolonged illness duration, obesity, and those presenting metabolic syndrome characteristics.

**Conclusion:**

The incidence of NAFLD among individuals with IBD notably exceeds that observed in the general populace. This heightened prevalence correlates with factors such as disease severity, metabolic risk profiles, and the impact of pharmacological interventions. Further research is needed to further elucidate these risk factors and establish screening recommendations.

## 1 Introduction

Non-alcoholic fatty liver disease has emerged as a significant health concern globally, with its incidence showing a swift rise worldwide (1). The estimated prevalence of NAFLD diagnosed through imaging is around 36.2% in the United States, 38.17% in Italy, 21.8% in Japan, and 56.9%in Canada, respectively, while the global prevalence is around 25.24% (2). Contributing factors to the development of NAFLD encompass a range of conditions including insulin resistance, obesity, along with metabolic syndrome and so on. Additionally, the use of specific medications like corticosteroids and methotrexate may also play a role in its onset (3). Approximately 10%–20% of NAFLD patients may develop non-alcoholic steatohepatitis (NASH) and progress to more severe conditions like fibrosis, cirrhosis, and complications like portal hypertension, hepatocellular carcinoma, and liver transplant requirements (4). Currently, NAFLD is the second leading reason for liver transplantation among adults, particularly women, and is expected to become the leading indication in the coming years (5–7).

The relationship between NAFLD and inflammatory bowel disease (IBD) is not well-understood, as data regarding the prevalence of NAFLD among IBD patients remains conflicting, and the underlying cause of the association between the two diseases is unclear (8). The interplay between these two conditions is intricate, shaped by a combination of environmental influences and genetic predispositions, with immune system imbalances being pivotal (9). NAFLD frequently coexists with diabetes, obesity, and metabolic syndrome, highlighting their interconnected nature; however, a subset of patients has “lean NAFLD” (10). While obesity is becoming more common in IBD patients, some with both IBD and NAFLD may belong to the “lean NAFLD” group, as research shows that underweight IBD patients can also have NAFLD (11). Furthermore, studies have suggested a connection between NAFLD phenotype and IBD disease severity, small bowel surgery history, and steroid use (12).

Previous studies exploring the link between IBD and NAFLD have largely relied on observational methods and have been constrained by the scope of single-institution research with limited participant numbers. The acknowledged restrictions have underscored the necessity for a comprehensive systematic review and meta-analysis. This approach aims to provide a more precise estimation of NAFLD prevalence among individuals with IBD and to discern the specific factors that correlate with NAFLD within this particular cohort. An in-depth and extensive analysis of this nature can yield significant understanding of the complex dynamics between IBD and NAFLD. It may pave the way for future studies and contribute to the formulation of more efficacious therapeutic approaches, ultimately benefiting patients who are afflicted with both disorders.

Understanding the relationship between IBD and NAFLD could provide important information for clinicians and researchers. For example, pinpointing shared risk factors or fundamental mechanisms might facilitate the crafting of targeted preventative measures or the innovation of groundbreaking treatments. In addition, this understanding could empower medical professionals to identify and track IBD patients who are more susceptible to developing NAFLD. This would allow for proactive measures to be taken, which may help in decelerating the advancement of liver complications. Moreover, these insights might illuminate the impact of lifestyle elements, including dietary habits and physical activity, on the management of both IBD and NAFLD conditions. Ultimately, a more comprehensive understanding of the relationship between these two diseases could contribute significantly to the improvement of overall quality of life for those patients affected by both IBD and NAFLD.

## 2 Materials and methods

The systematic review and meta-analysis were meticulously executed in adherence to the established protocols outlined by the Preferred Reporting Items for Systematic Reviews and Meta-Analyses (PRISMA). Each phase was carried out using the prescribed standard methodologies as recommended by the PRISMA guidelines.

Our research encompassed a comprehensive search on PubMed and MEDLINE databases, spanning from January 2014 to April 2024. We utilized a combination of indexing terms related to IBD and NAFLD, as detailed in Supplementary Table 2. The scope of our search was confined to English-language articles. Review, animal-based research, and studies that included subjects under 18 years of age were excluded. A single researcher conducted an initial review of the articles from the selected studies. Any discrepancies were addressed through mutual agreement or by consulting a third researcher. Our inclusion criteria were focused on studies that reported on patients diagnosed with IBD and those with NAFLD. Studies with small sample sizes, review articles, studies involving pediatric populations (under 18 years old), and animal studies were excluded. Our search strategy is shown in Supplementary Table 1 of the Supplementary material.

Our main objective was to ascertain the incidence of NAFLD among individuals with IBD and to evaluate if there was a notable variance when juxtaposed with the prevalence in the broader population. The data extraction process was executed solely by one researcher (Zhao) and subsequently validated by a second researcher (Wang), who conducted an independent verification. The data gathered encompassed a range of details including the study’s identity, participants’ age, gender, body mass index (BMI), the incidence of NAFLD among IBD patients, the study time of each study, and the geographical regions where the research was conducted. For all meta-analyses, the Cochrane I statistical approach was employed to evaluate the heterogeneity across studies. The meta and meta for packages in R version 4.4.0 (R Foundation for Statistical Computing) were used.

## 3 Results

**FIGURE 1:**
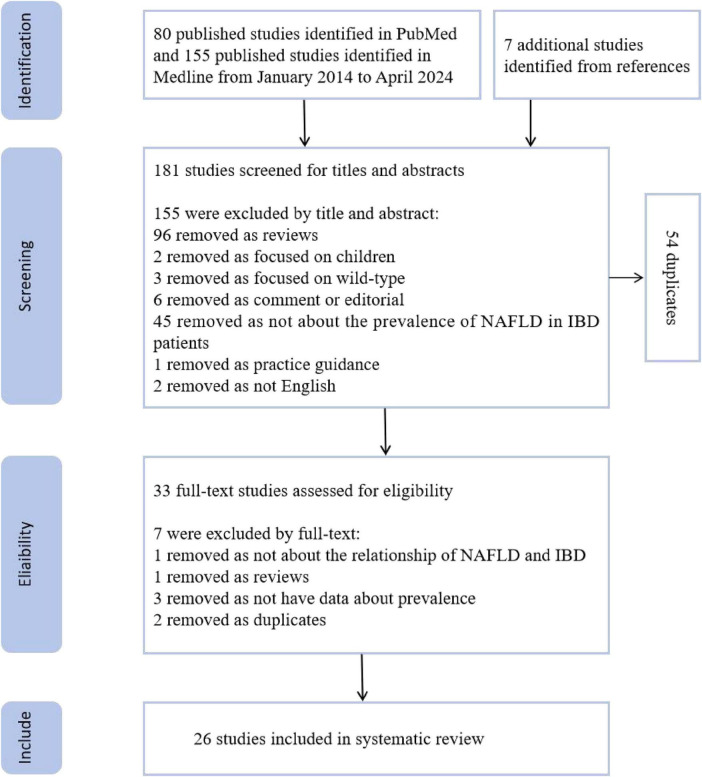
Search process. This research undertakes a systematic review methodology, concentrating on the relationship between inflammatory bowel disease (IBD) and non-alcoholic fatty liver disease (NAFLD). The primary objective is to determine the incidence of NAFLD within the IBD patients. Our initial step involved a search of the PubMed and Medline databases, encompassing literature from January 2014 to April 2024. A total of 235 published studies were identified, with 54 duplicates removed, leaving 181 studies for title and abstract screening. Following an initial assessment of titles and abstracts, 155 studies were deemed ineligible and subsequently excluded. Full-text eligibility assessment was conducted on the remaining 33 studies, leading to the further exclusion of seven studies. After a meticulous evaluation, the final systematic review was comprised of 26 studies that met all the inclusion criteria.

Through database searches, we retrieved 155 studies from Medline and 80 from PubMed, totaling 235 studies, of which 54 articles were identified as duplicates and removed. Thus, a total of 181 articles were screened in the first round, primarily through the examination of article titles and abstracts, resulting in the exclusion of 155 studies. Consequently, 26 articles remained after the first round. During the first round, an additional seven references were added through the articles’ bibliographies, all of which proceeded to the second round of screening, resulting in a total of 33 articles for the second round. The second round of screening involved reading the full text of the studies. After this round, seven articles were excluded, leaving a final total of 26 articles that met our pre-established criteria for inclusion in the study. The specific exclusion criteria are shown in Figure 1.

Table 1 summarizes the baseline characteristics of the 26 eligible studies’ abstracts or articles. The analysis included 429,550 patients (the sample size of the 26 studies ranged from 36 to 418,721 cases, with the smallest sample size being 36 cases and the largest being 418,721 cases). Among the 26 studies, age was not reported in 5 studies; among the remaining 21 studies that reported age, the average age of the patients was between 35 and 56 years old. Gender was not reported in nine studies; among the remaining 17 studies that reported gender, there were 199,728 males (accounting for 47% of the total number of people in the 17 studies that reported gender) and 226,585 females (accounting for 53% of the total number of people in the 17 studies that reported gender). Twenty-three studies did not differentiate between patients with Crohn’s disease and ulcerative colitis within the IBD population. Among the three studies that did differentiate, there were 655 cases of Crohn’s disease (accounting for 59% of the total number of people in the three studies that differentiated between Crohn’s disease and ulcerative colitis) and 456 cases of ulcerative colitis (accounting for 41% of the total number of people in the three studies that differentiated between Crohn’s disease and ulcerative colitis).

**TABLE 1:** Description of studies included in the systematic review and meta-analysis.

Study	References	Prevalence (95% CI)	Date	Country	NAFLD diagnostic methods
[1]	Sartini et al. (10)	33.6% (27.4%, 39.8%)	2012.3–2016.3	Italy	TE, abdominal ultrasound, APRI score
[2]	Adams et al. (11)	87.6% (79.6%, 95.6%)	2005.10–2018.7	Germany	MRI
[2]	Adams et al. (11)	21.5% (11.5%, 31.5%)	2005.10–2018.7	Germany	MRI
[3]	Glassner, Malaty, and Abraham (22)	13.3% (10.1%, 16.5%)	2015.1–2016.4	United States	CT, abdominal ultrasound, APRI score
[4]	Martínez-Domínguez et al. (23)	45% (41.8%, 48%)	2020.10–2021.10	Spain	Abdominal ultrasound, TE and laboratory tests
[5]	Principi et al. 2018 (24)	28% (24,6%, 31.4%)	2015.12–2016.7	United States	Liver ultrasound
[6]	Hoffmann et al. (25)	48% (43.3%, 52.7%)	2017.5.31–2018.5.31	Germany	Liver ultrasound
[6]	Hoffmann et al. (25)	44% (37.9%, 50.1%)	2017.5.31–2018.5.31	Germany	Liver ultrasound
[7]	Saroli Palumbo et al. (26)	32.8% (28.1%, 37.5%)	–	Canada	TE and CAP
[8]	van Lingen et al. (27)	40% (30.1%, 49%)	2017.6–2018.2	Netherlands	TE and CAP
[9]	Ritaccio et al. (28)	12.4% (7.9%, 16.9%)	2007–2017	United States	CT, ultrasound, MRI, liver biopsy
[10]	Bosch and Yeh (29)	26.9% (19.1%, 34.7%)	2001–2015	United States	TE and CAP
[11]	Kang et al. (30)	11.1% (8.2%,14%)	2004.1–2017.12	South Korea	CT
[12]	Hyun et al. (31)	16.7% (15.4%, 18%)	2005.11–2020.11	South Korea	HSI
[13]	Bessissow et al. (32)	33.6% (284%, 38.8%)	2006–2013	Canada	HSI
[14]	Zhang et al. (33)	38.4% (38.3%, 38.5%)	2006–2010	United Kingdom	TE
[15]	Sourianarayanane et al. (34)	8.2% (6.4%, 9.9%)	2009.1–2010.12	India	Radiological examination
[16]	Likhitsup, Dundulis, Ansari, El-Halawany, et al. (35)	44% (35.8%, 52.2%)	2009.1–2014.12	United States	CT
[16]	Likhitsup, Dundulis, Ansari, El-Halawany, et al. (35)	16% (9.9%, 22.1%)	2009.1–2014.12	United States	CT
[17]	Likhitsup, Dundulis, Ansari, Patibandla, et al. (36)	54% (43%, 65%)	–	United States	Abdominal ultrasound
[18]	Lopes et al. (37)	45.07% (33.5%, 56.6%)	–	Brazil	Abdominal ultrasound
[19]	Veltkamp et al. (38)	30.3% (19%, 41.1%)	2012–2016	Germany	Abdominal ultrasound
[20]	Trifan et al. (39)	46.3% (35.5%, 57.1%)	2021.9–2022.6	Romania	CAP
[21]	Balaban et al. (40)	30.5% (15.4%, 45.6%)	2015.11.1–2016.10.31	Romania	CAP
[22]	Fousekis et al. (41)	20% (14.7%, 25.3%)	1977–2016	Greece	Abdominal imaging examinations
[23]	Li, Lu, and Yu (42)	10.95% (5.7%, 16.2%)	2012.1.1–2016.5.1	China	Abdominal ultrasound
[24]	Magrì et al. (43)	40.4% (33.1%, 47.7%)	2016.12–2018.1	Italy	Abdominal ultrasound
[25]	Sagami et al. (44)	21.8% (17.1%, 26.5%)	2008.11–2014.10	Japan	Abdominal ultrasound
[26]	Simon et al. (45)	52% (47%, 57%)	2004	United States	CT

The table presents an detailed compilation of the studies featured in our systematic review and meta-analysis. It provides the detailed information, including the principal investigator’s details, the sample size, the prevalence with their respective 95% confidence intervals, study time, and the research country. CT, computed tomography; CAP, controlled attenuation parameter; HSI, hepatic steatosis index; TE, transient elastography.

**FIGURE 2:**
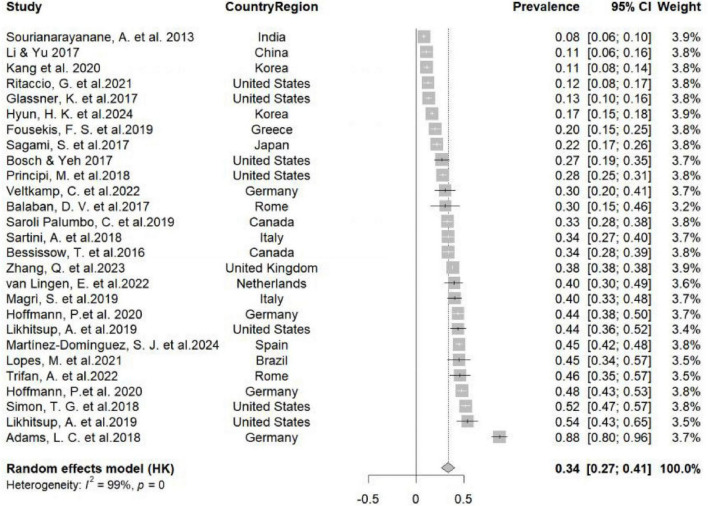
Forest plot of the prevalence of non-alcoholic fatty liver disease (NAFLD) from inflammatory bowel disease (IBD) patients in 26 studies. CI, confidence interval. This forest plot illustrates the aggregated findings from a meta-analysis, focusing on the incidence of NAFLD among patients with IBD from a wide range of geographical locations. The study included data from an extensive selection of countries, such as India, China, Korea, the United States, Greece, Japan, Germany and so on, showcasing a broad spectrum of global demographics. The heterogeneity assessment reveals a 99% heterogeneity rate among the studies in this meta-analysis. In this meta-analysis, the heterogeneity rate among the studies is 99%, indicating that there are significant differences in the design, samples, and methods of all included studies. The column denoting “weight” illustrates the proportional contribution of each individual study to the aggregate prevalence calculation.

Figure 2 shows the forest plot of NAFLD prevalence in IBD patients from 26 studies. Overall Prevalence of NAFLD in IBD Patients: Among 429,550 IBD patients from 26 studies, 163,647 had NAFLD, resulting in a pooled prevalence of 34% [95% Confidence Interval (CI), 27%–41%]. The heterogeneity was 99% (I^2^ = 99%). It is estimated that the pooled prevalence of NAFLD in the general global population is 25.2% (95% CI, 22.1%–28.7%) (2). Our meta-analysis reveals that the incidence of NAFLD among individuals with IBD is markedly elevated compared to that observed in the general population, with the difference being statistically significant.

**FIGURE 3:**
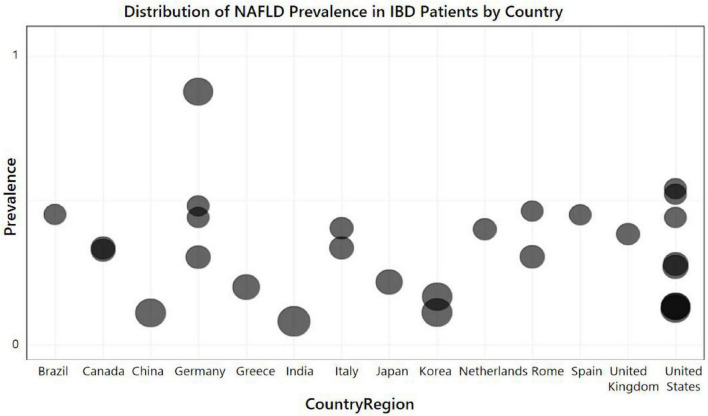
Distribution of non-alcoholic fatty liver disease (NAFLD) prevalence in inflammatory bowel disease (IBD) patients relative to the country of study. Each bubble in the chart represents data from a study corresponding to a particular country. The size of each circle represents the contribution of each individual study to the summary estimate, that is, the weight. This bubble chart aims to compare the prevalence of NAFLD across different countries and regions through a visual method.

Research on IBD patients has uncovered considerable disparities in the incidence of non-alcoholic fatty liver disease (NAFLD) across various nations which we employed a Bubble Chart to represent. Such variations in NAFLD prevalence among the countries surveyed could be attributed to a multitude of elements, such as genetic predispositions, dietary practices, and the standard of healthcare provision. The bubble plot showing the relationship between NAFLD prevalence in IBD patients and study countries is presented in Figure 3.

To explore the impact of national income levels on the prevalence of NAFLD in IBD patients, we conducted subgroup analyses by categorizing the included studies into high-, middle-, and low-income groups according to the World Bank criteria. The results showed that studies from high-income countries or regions constituted the vast majority (k = 24), with a pooled standardized mean difference (SMD) of 0.3522 (95% CI: 0.2812–0.4232), but with extremely high heterogeneity (I^2^ = 99.0%); there were fewer studies from middle-income countries (k = 2), with an SMD of 0.2760 (95% CI: −1.8911 to 2.4430) and similarly significant heterogeneity (I^2^ = 96.4%); and only one study from a low-income country (SMD = 0.0820, 95% CI: 0.0644–0.0996), for which heterogeneity could not be assessed.

## 4 Discussion

Our thorough review of 26 studies has revealed that the combined prevalence of NAFLD among patients with IBD stands at 34% (CI: 27%–41%). This rate is significantly higher than the 25.2% prevalence observed in the overall population (2). When examining regional data, we found the highest prevalence of NAFLD in IBD patients in 57 out of 65 cases (87.6%, 95% CI: 80%–97%) and the lowest in 76 of 928 cases (8.2%, 95% CI: 6.4%–9.9%) Factors linked to an increased risk of NAFLD in IBD patients include age, body mass index, presence of diabetes, and previous bowel resection.

Compared to the latest systematic reviews on the incidence of NAFLD among IBD patients, our research encompasses a more extensive patient base and demonstrates a significantly elevated rate of NAFLD in this group when contrasted with the wider population. Zou et al. identified 19 studies (5,620 patients) and found a pooled estimate of prevalence of NAFLD among IBD patients of 27.5% (95% CI, 20.7%–34.2%) (13). This prevalence is lower than that of our meta-analysis and more closely mirrors the overall prevalence of NAFLD among the general population (25.2%) (2). This difference might be accounted for by the difference in number of studies included (19 vs 26) and the difference in the number of patients (5,620 vs 429,550). Despite variations in reported prevalence, a consistent pattern of risk factors for NAFLD among IBD patients was identified, including older age, obesity, diabetes, a history of surgery related to IBD, and a prolonged duration of IBD symptoms. In a study conducted by Zou et al., chronic kidney disease (CKD), the use of methotrexate—a medication known to induce secondary hepatic steatosis—and hypertension were identified as substantial risk factors contributing to the onset of NAFLD in the IBD patient cohort (14).

Notably, NAFLD prevalence exhibited significant geographical disparities, with higher rates in Europe/North America versus Asia. Potential contributors include genetic predisposition, dietary patterns (e.g., Western diets associated with higher obesity rates), and healthcare resource availability affecting diagnostic practices. Further investigation into these regional differences is warranted to develop tailored prevention and management strategies for diverse IBD populations (15).

The inconsistency in diagnostic methods for non-alcoholic fatty liver disease (NAFLD) may lead to differences in results, as discussed below: Transient elastography (TE) combined with controlled attenuation parameter (CAP) has shown high sensitivity in multiple studies, enabling the detection of more NAFLD patients. For example, the prevalence of NAFLD detected by TE with CAP was 32.8% in study (7) and 30.5% in study (22), while the prevalence detected by ultrasound was generally lower [e.g., 28% in study (5)]. Therefore, TE combined with CAP may lead to a higher prevalence of NAFLD. In contrast, ultrasound has lower sensitivity and usually only detects more severe cases of fatty liver, thus resulting in a lower prevalence of NAFLD [e.g., 28% in study (5) and 48% in study (6)]. To reduce the impact of diagnostic method inconsistencies on results, it is recommended that future studies adopt more standardized diagnostic methods: TE combined with CAP, due to its high sensitivity and specificity, is recommended as the preferred method for NAFLD diagnosis, especially for large-scale epidemiological studies; MRI, although accurate, is costly and suitable for small-scale studies or as a validation standard; liver biopsy, as the gold standard, is recommended only for special cases or research validation due to its invasiveness (16, 17).

The high prevalence of NAFLD in patients with inflammatory bowel disease (IBD) may be related to various mechanisms, including metabolic factors, systemic inflammation, gut microbiota dysbiosis, and malnutrition. Although NAFLD is usually associated with obesity and metabolic syndrome, the pathogenesis of NAFLD in IBD patients has not been fully elucidated. Studies have shown that a higher BMI, diabetes, and intestinal resection may increase the risk, but the specific mechanisms still need to be explored—surgery may affect nutrient absorption or reflect a more severe inflammatory burden. In addition, systemic inflammation may drive the development of NAFLD by promoting hepatic fat accumulation and insulin resistance, while gut microbiota dysbiosis (such as abnormal short-chain fatty acid metabolism) and malnutrition (lack of protein or trace elements) may also impair liver metabolic function through the gut-liver axis (18). For high-risk patients (such as those with obesity, metabolic syndrome, or a history of surgery), it is recommended to use transient elastography (TE) combined with controlled attenuation parameter (CAP) for regular screening to assess the degree of hepatic steatosis and fibrosis. Future studies need to further clarify the interactions of these factors to optimize the prevention and treatment strategies for IBD-related NAFLD.

Although this study has proposed recommendations for NAFLD screening in high-risk IBD patients, the specific screening strategies still need to be further clarified. First, for IBD patients who are obese, have a longer disease duration, have metabolic syndrome (such as diabetes, hypertension), or have undergone intestinal surgery, regular NAFLD screening is recommended (19). Screening methods can include transient elastography (TE) combined with controlled attenuation parameter (CAP), which can simultaneously assess hepatic fat content and fibrosis degree, and has high sensitivity and specificity. For high-risk patients, it is recommended to undergo screening every 1–2 years to detect and manage NAFLD early and prevent its progression to more severe liver disease.

In addition, the impact of medications on NAFLD risk should not be overlooked. The use of corticosteroids is associated with weight gain and metabolic disorders, which may increase the risk of NAFLD. Therefore, for IBD patients on long-term corticosteroids, their metabolic indicators and liver function should be closely monitored, and dose reduction or switching to other immunosuppressants should be considered if necessary. On the other hand, anti-tumor necrosis factor (anti-TNF) drugs, while controlling IBD inflammation, may reduce the risk of NAFLD by improving systemic inflammatory status (20). However, anti-TNF drugs may also lead to weight gain, so the metabolic status and liver health of patients should be regularly assessed during their use (21).

Finally, the management of IBD combined with NAFLD requires the collaboration of a multidisciplinary team. Gastroenterologists should be responsible for the routine management of IBD, hepatologists should focus on the diagnosis and treatment of NAFLD, and dietitians can provide personalized dietary advice to help control weight and improve metabolic health. Collaboration among the multidisciplinary team not only helps optimize the overall treatment plan for patients but also ensures the best treatment outcomes for patients through regular communication and joint assessments in the management of both IBD and NAFLD.

Our study results have several limitations. Firstly, our results show significant differences in the prevalence of NAFLD among IBD patients, with heterogeneity as high as 99%, indicating that there are considerable differences in the design, samples, and methods of all included studies. The use of different diagnostic methods has led to significant differences in the results. One possible reason is the large sample size, which magnifies potential heterogeneity. Additionally, differences in patient characteristics, diagnostic criteria, and research settings across different studies may also contribute to the high heterogeneity. Future research should consider adopting more uniform diagnostic standards and methods to reduce heterogeneity and improve the comparability of results. Furthermore, only a few studies commented on the risk factors that might make IBD patients susceptible to developing NAFLD, and those that did provided data in heterogeneous formats and with varying statistical significance, limiting the generalizability. There is also significant variation in reporting patients’ medication exposure histories. It remains unclear whether any patients were simultaneously undergoing more than one treatment or whether the exposure was recent or past. Therefore, it is not possible to examine medication exposure as a risk factor for the development of NAFLD, making this a topic worth further investigation. The articles we used were limited to English, and these provided more data on North America and Europe than on Asia or other parts of the world.

Our research has discovered that NAFLD affects 34% of individuals with IBD, a rate significantly exceeding that of the general populace. We suggest that IBD patients with NAFLD should receive special attention during treatment, as they have an increased risk of liver damage from IBD therapies and poorer outcomes for those who are obese. Additional studies should investigate the benefits of screening for NAFLD in patients with risk factors such as advanced age, elevated BMI, diabetes mellitus, prolonged disease duration, and a history of surgical resection.

## Data Availability

The original contributions presented in the study are included in the article/Supplementary material, further inquiries can be directed to the corresponding author.
