# Supplementary material for: Prevalence of non-alcoholic fatty liver disease among inflammatory bowel disease patients: a systematic review and meta-analysis
Source: Front Med (Lausanne). 2025 Jul 16;12:1517462. doi: 10.3389/fmed.2025.1517462 (PMC12307432; doi:10.3389/fmed.2025.1517462)
Supplement: Supplementary file 1 [file Table_1.docx]

Supplementary

# **Supplemental Table 1: PUBMED、MEDLINE Search Strategy.**The table displays the literature search strategy for the study on "the prevalence of NAFLD in IBD patients." It lists two different databases: Pubmed and Medline, and provides detailed search formulas for each database.

| **Website** | **SEARCH STRATEGY** |
| --- | --- |
| **Pubmed** | (("inflammatory bowel diseases"[Title/Abstract] AND "non alcoholic fatty liver disease"[Title/Abstract]) OR "inflammatory bowel diseases"[MeSH Major Topic]) AND "non alcoholic fatty liver disease"[MeSH Major Topic] |
| **Medline** | ((((TI=(Inflammatory Bowel Diseases) OR AB=(Inflammatory Bowel Diseases)) AND (TI=(Non-alcoholic fatty liver disease) OR AB=(Non-alcoholic fatty liver disease)))) OR (MT=(Inflammatory Bowel Diseases)) AND MT=(Non-alcoholic fatty liver disease)) |

**Supplemental Table 2** Characteristics of Studies Involving Prevalence of NAFLD in IBD Patients. The table provides epidemiological data on NAFLD in IBD patients from different studies across various countries. The research spans multiple countries and medical centers, aiming to assess the prevalence of NAFLD in IBD patients and its associated risk factors. The table lists the sample size, prevalence of NAFLD, and the 95% confidence interval for each study, providing demographic information such as the age range, gender ratio, and BMI (Body Mass Index) of the study populations. It also considers a variety of potential risk factors, as well as the specific time frame for each study.

| **Study** | **SampleSize** | **prevalence** | **Men/ Women** | **Mean Age** | **Mean BMI** | **Start_date** | **Potential_Risk_**  **Factors** | **Country** |
| --- | --- | --- | --- | --- | --- | --- | --- | --- |
| [Sartini et al. 2018](https://paperpile.com/c/SVRpwM/tyWD) ^[1](https://paperpile.com/c/ve2M3s/4mvTD)^ | 223 | 33.6%  (27.4%,39.8%) | 92/130 | 53.04±12.16 | 29.43±5.38 | 2012/3-2016/3 | Severe steatosis、 extensive IBD | Italy |
| [Adams et al. 2018](https://paperpile.com/c/SVRpwM/N42C) ^[2](https://paperpile.com/c/ve2M3s/wbUmk)^ | 65 | 87.6%  (79.6%,95.6%) | 45/20 | 30.9±8.9 | 17.0 | 2005/10-2018 /7 |  | Germany |
| [Adams et al. 2018](https://paperpile.com/c/SVRpwM/N42C) ^[2](https://paperpile.com/c/ve2M3s/wbUmk)^ | Control:65 | 21.5%  (11.5%,31.5%) |  | 30.8±8.7 | 22.6 | 2005/10-2018 /7 |  | Germany |
| [Glassner, Malaty, and Abraham 2017](https://paperpile.com/c/SVRpwM/Gw08) ^[3](https://paperpile.com/c/ve2M3s/EVqU9)^ | 421 | 13.3%  (10.1%,16.5%) |  | 35.0±14.1 | 30.4±10.2 | 2015/1-2016/4 | Diabetes, Hypertension, Obesity, Hyperlipidemia | United States |
| [Martínez-Domínguez et al. 2024](https://paperpile.com/c/SVRpwM/6bP1) ^[4](https://paperpile.com/c/ve2M3s/Zng7b)^ | 911 | 45%  (41.8%,48.2%) | 449/462 | 48 |  | 2020/10-2021/10 | Advanced age at IBD diagnosis、 prolonged duration of IBD | ,Spain |
| [Principi et al. 2018](https://paperpile.com/c/SVRpwM/hoea) ^[5](https://paperpile.com/c/ve2M3s/BlA6b)^ | 654 | 28%  (24.6%,31.4%) | 334/320 | 45.08 ±16.5 | 24.53±4.81 | 2015/12-2016/7 | Metabolic syndrome, diabetes, GGT levels, fasting blood sugar, waist circumference. | United States |
| [Hoffmann et al.2020](https://paperpile.com/c/SVRpwM/Xiok) ^[6](https://paperpile.com/c/ve2M3s/sWitR)^ | 440 | 48%  (43.3%,52.7%) |  |  |  | 2017/5/31-2018/5/31 |  | Germany |
| [Hoffmann et al. 2020](https://paperpile.com/c/SVRpwM/Xiok) ^[6](https://paperpile.com/c/ve2M3s/sWitR)^ | 254 | 44%  (37.9%,50.1%) |  |  |  | 2017/5/31-2018/5/31 |  | Germany |
| [Saroli Palumbo et al. 2019](https://paperpile.com/c/SVRpwM/PjdU) ^[7](https://paperpile.com/c/ve2M3s/EWqrU)^ | 384 | 32.8%  (28.1%,37.5%) | 173/211 | 42.4 | 24.91 ±4.13 |  | Advanced age, higher body mass index, higher triglycerides. | Canada |
| [van Lingen et al. 2022](https://paperpile.com/c/SVRpwM/P1lP) ^[8](https://paperpile.com/c/ve2M3s/l6935)^ | 112 | 40%  (30.1%,49%) | 63/49 | 43±16.0 | 25.1±4.7 | 2017/6-2018/2 |  | Netherlands |
| [Ritaccio et al. 2021](https://paperpile.com/c/SVRpwM/xPIC) ^[9](https://paperpile.com/c/ve2M3s/d0NpI)^ | 207 | 12.4%  (7.9%,16.9%) | 96/111 | 50.6±13.6 | 29.5±8 | 2007-2017 |  | United States |
| [Bosch and Yeh 2017](https://paperpile.com/c/SVRpwM/WtNo) ^[10](https://paperpile.com/c/ve2M3s/IaPDt)^ | 123 | 26.9%  (19.1%,34.7%) |  |  |  | 2001-2015 |  | United States |
| [Kang et al. 2020](https://paperpile.com/c/SVRpwM/SL98) ^[11](https://paperpile.com/c/ve2M3s/MeLFH)^ | 443 | 11.1%  (8.2%,14%) |  |  |  | 2004/1-2017/12 |  | South Korea |
| [Hyun et al. 2024](https://paperpile.com/c/SVRpwM/360H) ^[12](https://paperpile.com/c/ve2M3s/FAHD9)^ | 3356 | 16.7%  (15.4%,18%) | 1,960/1396 | 44.1±15.8 | 25.2±4.0 | 2005/11-2020/11 | Pro-inflammatory state, insulin resistance, old age, metabolic syndrome. | South Korea |
| [Bessissow et al. 2016](https://paperpile.com/c/SVRpwM/Jwvv) ^[13](https://paperpile.com/c/ve2M3s/dQCDF)^ | 321 | 33.6%  (28.4%,38.8%) | 151/170 | 33.7 | 22.9 | 2006-2013 |  | Canada |
| [Zhang et al. 2023](https://paperpile.com/c/SVRpwM/5TVw) ^[14](https://paperpile.com/c/ve2M3s/gdZUN)^ | 418721 | 38.40%  (38.3%,38.5%) | 195678/223,043 | 56.22±8.11 |  | 2006-2010 |  | United Kingdom |
| [Sourianarayanane et al. 2013](https://paperpile.com/c/SVRpwM/RLDO) ^[15](https://paperpile.com/c/ve2M3s/yGmW0)^ | 928 | 8.2%  (6.4%,9.9%) |  | 46±13.3 |  | 2009/1-2010/12 | Hypertension, obesity, small intestine surgery, high BMI, obesity, hypertension, metabolic syndrome. | India |
| [Likhitsup, Dundulis, Ansari, El-Halawany, et al. 2019](https://paperpile.com/c/SVRpwM/FwdK) ^[16](https://paperpile.com/c/ve2M3s/R7GnP)^ | 70 | 44%  (35.8%,52.2%) | 31/39 | 38.6±15 | 26.6±6.9 | 2009/1-2014/12 |  | United States |
| [Likhitsup, Dundulis, Ansari, El-Halawany, et al. 2019](https://paperpile.com/c/SVRpwM/FwdK) ^[16](https://paperpile.com/c/ve2M3s/R7GnP)^ | Control:70 | 16%  (9.9%,22.1%) | 27/43 | 38.8±15 | 26.7±6.6 | 2009/1-2014/12 |  | United States |
| [Likhitsup, Dundulis, Ansari, Patibandla, et al. 2019](https://paperpile.com/c/SVRpwM/xP7Z) ^[17](https://paperpile.com/c/ve2M3s/QpYQU)^ | 80 | 54%  (43%,65%) | 44/36 | 42±15 | 26±5.7 |  | Male, high BMI, obesity. | United States |
| [Lopes et al. 2021](https://paperpile.com/c/SVRpwM/g9pU) ^[18](https://paperpile.com/c/ve2M3s/X1wEm)^ | 71 | 45.07%  (33.5%,56.6%) | 26/45 | 45.32±13.59 |  |  |  | São Paulo",Brazil |
| [Veltkamp et al. 2022](https://paperpile.com/c/SVRpwM/yOEF) ^[19](https://paperpile.com/c/ve2M3s/HJ9lD)^ | 132 | 30.3%  (19.5%,41.1%) | 57/75 | 42 | 23 | 2012-2016 | older age, high BMI, obesity | Germany |
| [Trifan et al. 2022](https://paperpile.com/c/SVRpwM/D0VG) ^[20](https://paperpile.com/c/ve2M3s/rNhNC)^ | 82 | 46.3%  (35.5%,57.1%) | 45/37 | 49±13 | 25.3±4.7 | 2021/9-2022/6 | Age、Body Mass Index (BMI)、Disease duration、C-reactive protein (CRP)、Fasting blood sugar、Type 2 Diabetes Mellitus (T2DM)、Triglycerides、Age at diagnosis、History of bowel resection | Romania |
| [Balaban et al. 2017](https://paperpile.com/c/SVRpwM/UTpX) ^[21](https://paperpile.com/c/ve2M3s/iYi72)^ | 36 | 30.5%  (15.4%,45.6%) | 17/19 | 43±13 | 23.55 | 2015/11/1-2016/10/31 |  | Romania |
| [Fousekis et al. 2019](https://paperpile.com/c/SVRpwM/XgnT) ^[22](https://paperpile.com/c/ve2M3s/oFpKT)^ | 220 | 20%  (14.7%,25.3%) | 134/86 | 39±17.4 | 1977 | 2016 |  | Greece |
| [Li, Lu, and Yu 2017](https://paperpile.com/c/SVRpwM/vS0T) ^[23](https://paperpile.com/c/ve2M3s/xOVTx)^ | 137 | 10.95%  (5.7%,16.2%) | 90/47 | 36.2±12.62 | 19.26±2.99 | 2012/1/1-2016/5/1 |  | China |
| [Magrì et al. 2019](https://paperpile.com/c/SVRpwM/SU0D) ^[24](https://paperpile.com/c/ve2M3s/b35JV)^ | 178 | 40.4%  (33.1%,47.7%) |  | 53.9±12.9 | 26.54 | 2016/12-2018/1 | Male、Elderly age | Italy |
| [Sagami et al. 2017](https://paperpile.com/c/SVRpwM/HHfO) ^[25](https://paperpile.com/c/ve2M3s/dYQ77)^ | 303 | 21.8%  (17.1%,26.5%) |  |  |  | 2008/11-2014/10 |  | Japan |
| [Simon et al. 2018](https://paperpile.com/c/SVRpwM/IZuz) ^[26](https://paperpile.com/c/ve2M3s/V3OsQ)^ | 462 | 52%  (47.3%,56.7%) | 216/246 | 40±15 | 25.4±5.0 | 2004 | Age、Diabetes、Value-Added Tax Quartile、PNPLA3 (G) Genotype | United States |

**References**

1. Sartini, A. et al. Non-alcoholic fatty liver disease phenotypes in patients with inflammatory bowel disease. Cell Death Dis. 9, 87 (2018).

2. Adams, L. C. et al. Non-alcoholic fatty liver disease in underweight patients with inflammatory bowel disease: A case-control study. PLoS One 13, e0206450 (2018).

3. Glassner, K., Malaty, H. M. & Abraham, B. P. Epidemiology and Risk Factors of Nonalcoholic Fatty Liver Disease Among Patients with Inflammatory Bowel Disease. Inflamm. Bowel Dis. 23, 998–1003 (2017).

4. Martínez-Domínguez, S. J. et al. Crohn´s disease is an independent risk factor for liver fibrosis in patients with inflammatory bowel disease and non-alcoholic fatty liver disease. Eur. J. Intern. Med. 120, 99–106 (2024).

5. Principi, M. et al. Nonalcoholic Fatty Liver Disease in Inflammatory Bowel Disease: Prevalence and Risk Factors. Inflamm. Bowel Dis. 24, 1589–1596 (2018).

6. Hoffmann, P., Jung, V., Behnisch, R. & Gauss, A. Prevalence and risk factors of nonalcoholic fatty liver disease in patients with inflammatory bowel diseases: A cross-sectional and longitudinal analysis. World J. Gastroenterol. 26, 7367–7381 (2020).

7. Saroli Palumbo, C. et al. Screening for Nonalcoholic Fatty Liver Disease in Inflammatory Bowel Diseases: A Cohort Study Using Transient Elastography. Inflamm. Bowel Dis. 25, 124–133 (2019).

8. van Lingen, E. et al. Disease activity in inflammatory bowel disease patients is associated with increased liver fat content and liver fibrosis during follow-up. Int. J. Colorectal Dis. 37, 349–356 (2022).

9. Ritaccio, G. et al. Nonalcoholic Fatty Liver Disease Is Common in IBD Patients However Progression to Hepatic Fibrosis by Noninvasive Markers Is Rare. Dig. Dis. Sci. 66, 3186–3191 (2021).

10. Bosch, D. E. & Yeh, M. M. Primary sclerosing cholangitis is protective against nonalcoholic fatty liver disease in inflammatory bowel disease. Hum. Pathol. 69, 55–62 (2017).

11. Kang, M. K., Kim, K. O., Kim, M. C., Park, J. G. & Jang, B. I. Sarcopenia Is a New Risk Factor of Nonalcoholic Fatty Liver Disease in Patients with Inflammatory Bowel Disease. Dig. Dis. 38, 507–514 (2020).

12. Hyun, H. K. et al. Hepatic Steatosis but Not Fibrosis Is Independently Associated with Poor Outcomes in Patients with Inflammatory Bowel Disease. Gut Liver 18, 294–304 (2024).

13. Bessissow, T. et al. Incidence and Predictors of Nonalcoholic Fatty Liver Disease by Serum Biomarkers in Patients with Inflammatory Bowel Disease. Inflamm. Bowel Dis. 22, 1937–1944 (2016).

14. Zhang, Q. et al. Non-alcoholic fatty liver degree and long-term risk of incident inflammatory bowel disease: A large-scale prospective cohort study. Chin. Med. J. (2023) doi:10.1097/CM9.0000000000002859.

15. Sourianarayanane, A. et al. Risk factors of non-alcoholic fatty liver disease in patients with inflammatory bowel disease. J. Crohns. Colitis 7, e279–85 (2013).

16. Likhitsup, A. et al. Prevalence of non-alcoholic fatty liver disease on computed tomography in patients with inflammatory bowel disease visiting an emergency department. Ann. Gastroenterol. Hepatol. 32, 283–286 (2019).

17. Likhitsup, A. et al. High prevalence of non-alcoholic fatty liver disease in patients with inflammatory bowel disease receiving anti-tumor necrosis factor therapy. Ann. Gastroenterol. Hepatol. 32, 463–468 (2019).

18. Lopes, M. et al. P045 Prevalence of non-alcoholic fatty liver disease (NAFLD) in patients with inflammatory bowel disease (IBD) in a Brazilian public healthcare clinic. Am. J. Gastroenterol. 116, S11–S12 (2021).

19. Veltkamp, C. et al. Hepatic Steatosis and Fibrosis in Chronic Inflammatory Bowel Disease. J. Clin. Med. Res. 11, (2022).

20. Trifan, A. et al. Screening for Liver Steatosis and Fibrosis in Patients with Inflammatory Bowel Disease Using Vibration Controlled Transient Elastography with Controlled Attenuation Parameter. J. Clin. Med. Res. 11, (2022).

21. Balaban, D. V. et al. P323 Fatty liver assessment in inflammatory bowel disease patients using controlled attenuation parameter. J. Crohns. Colitis 11, S240–S241 (2017).

22. Fousekis, F. S. et al. Hepatobiliary and pancreatic manifestations in inflammatory bowel diseases: a referral center study. BMC Gastroenterol. 19, 48 (2019).

23. Li, D., Lu, C. & Yu, C. High incidence of non-alcoholic fatty liver disease in patients with Crohn’s disease but not ulcerative colitis. Int. J. Clin. Exp. Pathol. 10, 10633–10639 (2017).

24. Magrì, S. et al. Nonalcoholic fatty liver disease in patients with inflammatory bowel disease: Beyond the natural history. World J. Gastroenterol. 25, 5676–5686 (2019).

25. Sagami, S. et al. Significance of non-alcoholic fatty liver disease in Crohn’s disease: A retrospective cohort study. Hepatol. Res. 47, 872–881 (2017).

26. Simon, T. G. et al. IRGM Gene Variants Modify the Relationship Between Visceral Adipose Tissue and NAFLD in Patients With Crohn’s Disease. Inflamm. Bowel Dis. 24, 2247–2257 (2018).
